# Description and Molecular Characterization of Two Species of Avian Blood Parasites, with Remarks on Circadian Rhythms of Avian Haematozoa Infections

**DOI:** 10.3390/ani11123490

**Published:** 2021-12-07

**Authors:** Carolina Romeiro Fernandes Chagas, Rasa Binkienė, Gediminas Valkiūnas

**Affiliations:** Nature Research Centre, Akademijos 2, 08412 Vilnius, Lithuania; rasa.binkiene@gamtc.lt (R.B.); gediminas.valkiunas@gamtc.lt (G.V.)

**Keywords:** new blood parasite species, birds, co-infection, *Plasmodium*, *Haemoproteus*, *Leucocytozoon*, *Trypanosoma*, *Lankesterella*, microfilariae, *Splendidofilaria*

## Abstract

**Simple Summary:**

The diversity of avian blood parasites is remarkable, and they are often found in co-infections, which is a challenge for wildlife parasitology research. Co-infections not only make parasite morphological and molecular identification difficult, but also might influence the infection dynamics and disease manifestation. This study investigated blood parasites infections in a Eurasian blackbird (*Turdus merula*) and a Song thrush (*Turdus philomelos*). A new *Lankesterella* species was found and described. The molecular characterization of this parasites as well as *Splendidofilaria mavis*, an avian filarioid nematode, was developed. As several blood infections were present in co-infection, we used this opportunity to investigate the daily changes in the parasite intensity seen in the blood of these birds. A peak of the *Plasmodium matutinum* parasitemia was seen during the daytime. *Leucocytozoon* spp. showed some parasitemia peaks close to the evening and night. *Trypanosoma* spp. and *S. mavis* parasitemia increased during the night. Data about daily variation of *Haemoproteus, Lankesterella*, and *S. mavis* parasites were obtained for the first time. No pattern in parasitemia dynamics was identified in *Haemoproteus* and *Lankesterella* infections.

**Abstract:**

Avian blood parasites are remarkably diverse and frequently occur in co-infections, which predominate in wildlife. This makes wildlife pathogen research challenging, particularly if they belong to closely related groups, resulting in diagnostic problems and poor knowledge about such infections as well as the patterns of their co-occurrence and interactions. This is particularly true due to the periodicity (circadian rhythms) of parasitemia, which means that different parasitemia and parasite stages might be found throughout the day. We analysed blood samples from a Eurasian blackbird (*Turdus merula*) and a Song thrush (*Turdus philomelos*). This study aimed to describe a new avian *Lankesterella* species and molecularly characterize and redescribe *Splendidofilaria mavis,* a common avian filarioid nematode. Additionally, it was possible to investigate the circadian rhythms of the avian blood parasites belonging to *Plasmodium*, *Haemoproteus*, *Leucocytozoon*, and *Trypanosoma,* which occurred in co-infection in the same avian host individuals. Different circadian rhythms were seen in different parasites, with *Plasmodium* sp. peaks occurring at midday, *Leucocytozoon* spp. peaks mainly during the evening and night, and *Trypanosoma* spp. and microfilariae peaks at midnight. No periodicity was seen in *Haemoproteus* and *Lankesterella* species infections. The time of parasitemia peaks most likely coincides with the time of vectors’ activity, and this should be beneficial for transmission. Knowledge about the circadian rhythms is needed for better understanding patterns in host-parasite interactions and disease transmission.

## 1. Introduction

Avian blood parasites are an ecological group that is composed of protists and helminths which have been markedly unevenly studied [1]. For example, some haemosporidian parasites (*Plasmodium* and *Haemoproteus*) are extensively studied due to their close relationships to human malaria agents [2,3], while other avian blood pathogens such as *Lankesterella* and filarioid nematodes remain poorly investigated and insufficient knowledge is available even about basic stages of their life cycles [4,5]. One of the main challenges that is faced by researchers in wildlife parasitology is the presence of co-infections which are commonly observed in naturally infected wild birds [6,7,8,9] and even predominate in some bird populations [10]. As a result, both molecular diagnostics that use general primers [11,12] and morphological identifications might be remarkably challenging [13,14]. Moreover, blood parasite infections are markedly dynamic, resulting in variation of the parasitemia in the same host individuals not only in a long-term perspective but also during the same day [3,15,16,17].

The daily rhythms of biological activity, also known as circadian rhythms, were reported in numerous prokaryotic and eukaryotic organisms, including parasites. The periodicity of biological activity might trigger immunological responses and hormone levels that allow the organisms to anticipate changes in their external environment, resulting in fitness advantages [15,18,19,20]. This is a particularly sensitive issue for parasites which need not only to avoid the host immunological system, but also to increase their chances of being transmitted to the new hosts. The optimization of transmission is particularly important for vector-transmitted parasites whose survival needs to be adjusted for the accession of specific vectors at a particular stage of the pathogen life cycle when invasive stages are available [15,18,19,20]. For example, a clear circadian rhythm was observed in the *Wuchereria bancrofti*, a filarioid nematode of humans. This parasite is transmitted mainly by *Culex* mosquitoes which are active during the night when parasitemia of microfilariae increases as well [21]. Parasites with a direct life cycle of transmission also benefit from circadian rhythms. For example, the invasive stages (oocysts) of avian *Isospora* species were massively released in the environment only in the late afternoon. This minimized direct exposure to sunlight, reducing desiccation and destruction by the solar ultraviolet radiation of the oocysts and, consequently, contributed to their better survival [22].

Knowledge about parasite diversity and parasitic diseases is essential in a changing world not only regarding human and domestic animal health, but also for a better understanding of wildlife disease epidemiology and evolutionary patterns [23]. This is true concerning poorly investigated parasite circadian rhythms, which a better understanding might show new directions for the development of prophylactic measures for diseases control and prevention. This might be helpful to improve research on parasite biology, gene expression, experimental investigations, pharmacological studies, and finally contribute to the development of measures to prevent new life-threatening infections to humans. However, the knowledge about many groups of wildlife parasites remains sparse and focused mainly on parasite species that are important in human and veterinary medicine or are agents of zoonotic diseases. This study aimed to contribute with new knowledge about common, but neglected, avian blood parasites. The main tasks were (i) to describe a new species of *Lankesterella* infecting a Eurasian blackbird (*Turdus merula*)*,* and (ii) molecularly characterize *Splendidofilaria mavis*, a filarioid nematode infecting the Eurasian blackbird and a Song thrush (*Turdus philomelos*). Because both studied avian hosts were co-infected with several species of blood parasites, we used this opportunity to investigate the circadian rhythms of these parasites as well as other pathogens that were found in co-infection and belong to *Plasmodium, Haemoproteus, Leucocytozoon*, and *Trypanosoma* genera.

## 2. Materials and Methods

### 2.1. Selection of Donor Birds and Examination of Blood Films

The study was conducted in Ventės Ragas Ornithological Station, Lithuania (55°20′28.1″ N, 21°11′25.3″ E) during May 2019. To select donors, wild birds were caught using funnel traps, mist nets, and ‘zigzag’ traps. About 50 µL of blood was taken by puncturing the vena ulnaris cutanea using heparinized capillary tubes. A few drops of blood were used to prepare blood films and a few drops of blood were stored in SET buffer (0.05 M tris, 0.15 M NaCl, 0.5 M EDTA, pH 8.0) for molecular analysis. The remaining blood was kept in the capillary tubes and processed using the buffy coat method [5]. Briefly, the blood samples were collected in heparinized capillary tubes, sealed with plasticine at one end, and centrifugated for 5 min. at 10,000 rpm. Next, the capillary tubes were placed above a glass slide and the buffy coat area was examined under low magnification (100×) in a microscope. Then, wet preparations were prepared by breaking the capillary tubes about 1 mm below the buffy coat layer. The buffy coat and plasma were transferred to a glass slide and screened using a microscope under 400× magnification.

The blood films were air-dried using a battery-operated fan, fixed by immersion in absolute methanol for 1 s, dried at room temperature, and stained with a 10% Giemsa solution for 1 h [3]. Microscopic examination of the blood films consisted of screening each slide for 15–20 min at low magnification (400×) and at least 100 fields were examined at high magnification (1000×) using an Olympus CX23 light microscope (Olympus, Tokyo, Japan). A total of two birds were selected for this study. These were: a Eurasian blackbird (*Turdus merula*) that was infected with *Plasmodium, Haemoproteus, Leucocytozoon, Trypanosoma, Lankesterella* and microfilaria of filarioid nematode, and a Song thrush (*Turdus philomelos*) that was infected with *Haemoproteus, Leucocytozoon*, and microfilaria of filarioid nematode.

Images of the haemosporidians, *Trypanosoma*, and *Lankesterella* parasites were collected using an Olympus BX41 light microscope that was equipped with an Olympus DP-12 digital camera and the Olympus DP-Soft v. 3.2 imaging software (Olympus, Tokyo, Japan). These images were used for illustration of the morphology and measurement of the described parasites. Statistical analyses were carried out using ‘R studio’ v.3.4.3. Voucher preparations of *Plasmodium matutinum* lineage pLINN1 (accession numbers NS49369 and NS49370), *Haemoproteus minutus* hTURDUS2 (NS49371 and NS49372), *Leucocytozoon* spp. lNEVE01 and lTURMER15 (NS49373 and NS49374), *Lankesterella bivacuolata* n. sp. LanTurd1 (NS49367 and NS49368)*, Trypanosoma avium* (NS49375 and NS49376), *Trypanosoma everetti* group (NS49377 and NS49378)*, Haemoproteus homominutus* unknown lineage (NS49379 and NS49380), and *Leucocytozoon* sp. lSTUR1 (NS49381 and NS49382) were deposited in Nature Research Centre. 

### 2.2. Circadian Rhythm Investigation

To follow parasitemia changes during a day, the selected birds were kept in cages with water and food *ad libitum*, at room temperature (~22 °C), and with a natural light:dark photoperiod (about 17:7 h). The Eurasian blackbird was observed for five days and the Song thrush for four days. Blood was taken for microscopic and molecular analysis from both experimental birds every 6 h (6 h, 12 h, 18 h and 24 h) using the same methodology as described in Section 2.1. At the end of the observation period, both birds were euthanized and dissected; adult worms of filarioid nematodes were collected for species identification (see description below).

Parasitemia intensity was determined using different methods depending on the parasite genus, as shown in Table 1. The blood stages were classified according to the certain parasite life cycle during the analysis of the data for determination of circadian rhythms. In *Plasmodium* parasites these were young and mature meronts, and young and mature gametocytes; in *Haemoproteus* and *Leucocytozoon,* young and mature gametocytes were examined separately as well (see Section 3.1). In *Trypanosoma* (see Section 3.1)*, Lankesterella* (see Section 3.2) and microfilariae (see Section 3.3) infections, only one blood stage was observed and analysed, i.e., trypomastigotes, sporozoites, and microfilariae, respectively.

### 2.3. Collection of Filarioid Worms

After the investigation of the circadian rhythm, the infected birds were euthanized and immediately dissected. The dissected organs and other tissues were placed in Petri dishes containing 0.9% saline solution and the parasites were recovered using dissecting needles and pipettes under a stereomicroscope (MБС-9). The subcutaneous tissues, joints of the leg and wings, brain, eyes, heart, connective tissues of trachea and oesophagus, lungs, air sacs, and body cavities for adult filarioid nematodes were examined according to Binkienė et al. [4]. Adult alive nematodes were examined under the microscope in 0.9% saline solution and then stored in 70% ethanol. For light-microscope examination, they were cleared with glycerine [28]. Blood from the liver and lungs was collected and stored in absolute ethanol for molecular analysis. Blood films from the liver and lungs were also prepared from these organs; these preparations were fixed, stained, and examined as the blood films (see description above). All blood films and preparations of nematodes were examined using an Olympus BX 51 light microscope equipped with differential interference contrast optics and a digital image analysis system (DeltaOptical DLTCam Viewer 3.7.8301). The identification of the parasite species was done using keys and species descriptions [29,30,31]. Voucher parasite specimens were deposited in the Nature Research Centre, Vilnius, Lithuania (accession numbers 1082–1106).

### 2.4. DNA Extraction, PCR and Molecular Identification of Parasites

Two methodologies were used for DNA extraction. For peripheral blood samples, the DNA was extracted using an ammonium acetate protocol [32]. For the blood samples that were obtained from the organs and adult nematodes, DNA extraction was done according to Stunžėnas et al. [33] with a slight modification according to Petkevičiūtė et al. [34]; mainly, the samples were well dried for 15 min on a sterile microscope glass slide before the DNA extraction. Polymerase chain reaction (PCR) protocols were applied for the determination of parasite species and/or their genetic lineages using general primers that were specific for each parasite group (Table 1). To control for false amplifications, all PCR reactions were run using one negative (ultra-pure water) and one positive (according to the targeted parasite) control.

The amplification success was determined using electrophoresis in a 2% agarose gel. Positive PCR products were precipitated and sequenced from both strands using the Big Dye Terminator V3.1 Cycle Sequencing Kit (Thermo Fisher Scientific, Vilnius, Lithuania) and ABI PRISM™ 3100 capillary sequencing robot (Applied Biosystems, Foster City, CA, USA). The obtained sequences were edited and aligned using BioEdit software [35] to create a consensus sequence. The presence of double peaks in electropherograms was considered as an indication of a co-infection with the parasite of target group. The obtained consensus sequences were aligned using BLAST (Basic Local Alignment Search Tool) and compared with sequences from MalAvi database (http://mbio-serv2.mbioekol.lu.se/Malavi/blast.html, accessed on 12 October 2021, for haemosporidian parasites) and GenBank database (http//www.ncbi.nlm.nih.gov/BLAST, accessed on 12 October 2021, for other blood parasites). For filarioid nematodes, the sequences that were obtained from the microfilaria and the adult worms were compared with each other to prove that they belonged to the same species. All detected parasite DNA sequences were deposited in GenBank: *P. matutinum* pLINN1 (OK646331), *H. minutus* hTURDUS2 (OK646332), *H. asymmetricus* hTUPHI01 (OK721103), *Leucocytozoon* sp. lNEVE01 (OK646333), *Leucocytozoon* sp. lTURMER15 (OK646334), *Leucocytozoon* sp. lSTUR1 (OK646335), *L. bivacuolata* n. sp. (OK644714)*, T. avium* (OK605056)*, S. mavis 28S* (OK644715-OK644718), and *S. mavis coxI* (OK631737-OK391740).

### 2.5. Plylogenetic Analysis 

Phylogenetic analysis was done to determine the relationships of the new *Lankesterella* species and the newly molecularly characterized *Splendidofilaria mavis* with other *Lankesterella* parasites and filarioid nematodes, respectively. 

A Bayesian phylogeny was constructed for *Lankesterella* parasites using *18S* rRNA gene partial sequences. The alignment consisted of sequences from 12 closely related parasite genera, including the one that was obtained in this study. We used the same DNA sequences as described by Chagas et al. [26]. Sequences of haemosporidian parasites (*Plasmodium*, *Haemoproteus*, and *Leucocytozoon* species) were used as an outgroup.

A Bayesian phylogeny was constructed using *S. mavis* nuclear *28S* gene partial sequence and software MrBayes version 3.2 [36]. Even though two genes were used for the identification of *S. mavis* (Table 1), only *28S* was used in the phylogenetic inference because it seemed to be the best marker for filarioid nematode species identification [4]. The alignment consisted of 26 DNA sequences, including the ones that were obtained in this study. A sequence of *Ascaridia galli* was used as an outgroup.

For both *Lankesterella* sp. and *S. mavis* phylogenetic inference, the sequences were aligned using MAFFT software [37]. The general time-reversible (GTR) was selected by the software MrModelTest2 software [38] as the best-fit substitution model. The run was conducted with four chains and with a sampling frequency of every 100th generation over 3 million generations. We discarded 25% of the trees as ‘burn-in’. The remaining trees were used to construct a consensus tree. The phylogenetic trees were visualized using FigTree 1.4 software [39].

## 3. Results

### 3.1. Parasite Morphological and Molecular Identification

#### 3.1.1. Eurasian Blackbird Infections

**Figure 1:**
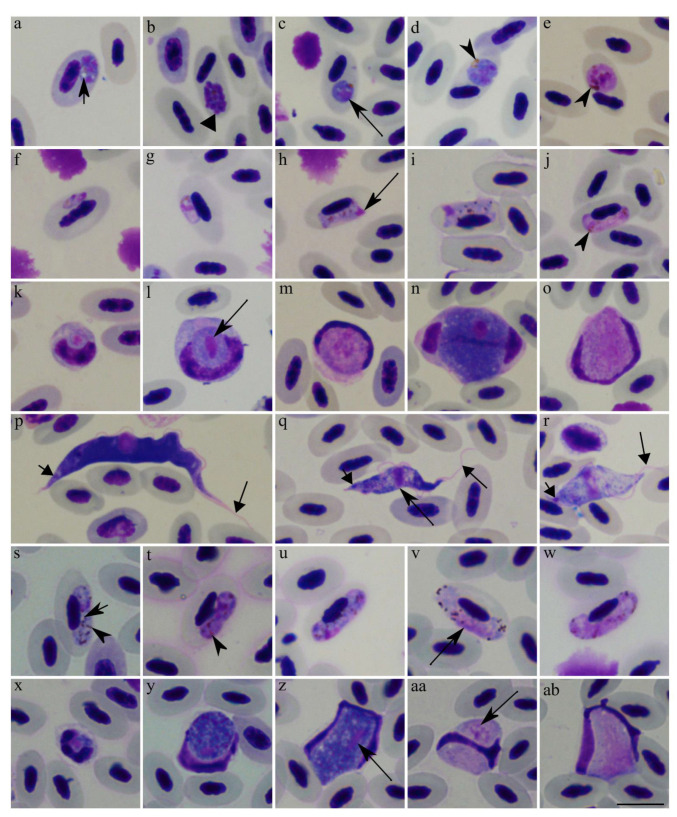
Blood parasites that were found in a Eurasian blackbird (*Turdus merula*) (**a**–**r**) and a Song thrush (*Turdus philomelos*) (**s**–**ab**). *Plasmodium matutinum* (cytochrome *b* lineage pLINN1): growing (**a**) and mature (**b**) meronts, growing (**c**) and mature (**d**) macrogametocytes, and microgametocyte (**e**). *Haemoproteus minutus* (hTURDUS2): young gametocytes (**f**,**g**), growing macrogametocyte (**h**), mature macrogametocyte (**i**) and microgametocyte (**j**). *Leucocytozoon* sp. (unknown lineage): young (**k**) and growing (**l**,**m**) gametocytes, mature macrogametocyte (**n**) and microgametocyte (**o**). *Trypanosoma avium* (**p**) and *Trypanosoma everetti* (**q**,**r**) group parasites. *Haemoproteus homominutus* (unknown lineage): growing gametocytes (**s**,**t**), and mature macrogametocyte (**u**) and microgametocytes (**v**,**w**). *Leucocytozoon* sp. (lSTUR1): young (**x**) and advanced growing (**y**) gametocytes, mature macrogametocyte (**z**) and microgametocytes (**aa**,**ab**). Triangle arrowhead (►), merozoite. Short-barbed arrow (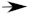), vacuole. Arrowhead (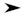), pigment granules. Long arrow (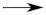), parasite nucleus. Short triangle arrow (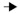), kinetoplast. Long triangle arrow (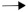), flagellum. Scale bar: 10 µm. Methanol fixed and Giemsa-stained blood films.

The Eurasian blackbird was infected with *Plasmodium matutinum (cytb* lineage pLINN1) (Figure 1a–e); *Haemoproteus minutus* (hTURDUS2) (Figure 1f–j)*; Leucocytozoon* spp. (lNEVE01 and lTURMER15) (Figure 1k–o)*; Lankesterella bivacuolata* n. sp. (*18S* lineage LanTurd1, see description in Section 3.2)*; Trypanosoma avium* (Figure 1p); and *Trypanosoma everetti* group parasites (Figure 1q,r)*,* and *Splendidofilaria mavis* (morphological features presented in Section 3.3). Parasite identification was supported by morphological and/or DNA barcoding methods.

The identification of the lineage pLINN1 of *P. matutinum* was confirmed in blood films due to the combination of the following characteristics: the presence of large vacuoles in the erythrocytic meronts (Figure 1a), which possess between 10 and 24 merozoites (Figure 1b) and the presence of roundish fully grown gametocytes (Figure 1d,e) possessing relatively small pigment granules. A detailed description and molecular characterization of this parasite were developed by Valkiūnas et al. [40].

*Haemoproteus minutus* (hTURDUS2) was readily distinguished morphologically in blood films due to (i) the presence of tiny fully grown gametocytes which do not fill the erythrocytes up to their poles and possess a few pigment granules (Figure 1i,j); (ii) the availability of growing gametocytes that do not touch erythrocyte envelope (Figure 1h); (iii) the pale staining of macrogametocytes (compare Figure 1u with Figure 1v); (iv) the absence of one readily distinguishable vacuole in the majority of the macrogametocytes, a feature of *Haemoproteus homominutus* (see description below); and (v) the predominant terminal position of nuclei in macrogametocytes (Figure 1h,i). For a detailed parasite description, see [3].

*Leucocytozoon* species identification was difficult. The observed mature gametocytes and host-cell nuclei were similar to *Leucocytozoon dubreuili* (Figure 1k–o), a common parasite of *Turdus* birds. Mainly, the gametocytes developed in roundish host cells, whose nuclei were more or less dumbbell-shaped with thickening at both ends and with the nuclei extending more than half of the circumference of gametocytes [3]. However, even though we obtained two DNA sequences of *Leucocytozoon* parasites, it was impossible to morphologically link them to certain species due to co-infection of morphologically similar gametocytes. The circadian rhythms’ analysis was done on the *Leucocytozoon* parasite genus level.

Morphotypes of the reported *Trypanosoma* species belonged to *Trypanosoma avium* and *Trypanosoma everetti* group (Figure 1p–r). These parasites were readily distinguishable from each other, particularly due to their different size and different position of kinetoplasts in trypomastigotes (compare Figure 1p with Figure 1q,r). However, even though it was possible to obtain *T. avium* DNA sequence, all the detected electropherograms still showed co-infections. As a result, certain linkage of DNA sequence information and morphotypes was impossible and the DNA sequences of all seen *Trypanosoma* species infecting this bird could not be determined. Since trypomastigotes of *T. avium* and *T. everetti* were readily distinguishable in blood films, we used this opportunity to proceed with the circadian rhythms’ analysis using only morphological criteria. For detailed descriptions of both *Trypanosoma* parasites, see Baker [41] and Molyneux [42].

In all, a total of 15 specimens of adult worms were recovered in the ankle joints of the Eurasian blackbird. The morphology of these nematodes corresponded to the description of *Splendidofilaria mavis* [30]. 

#### 3.1.2. Song Thrush Infections

The Song thrush was infected with *Haemoproteus homominutus* (unknown lineage) (Figure 1s–w), *Leucocytozoon* sp. (lSTUR1) (Figure 1x–ab) and *Splendidofilaria mavis* (morphological features presented in Section 3.3). Parasite identification was supported by morphological and/or DNA barcoding methods.

The *Haemoproteus* that was infecting this bird was morphologically identical to *Haemoproteus homominutus* (Figure 1s–w) due to (i) the presence of small fully grown gametocytes which fill the erythrocytes up to their poles (Figure 1v,w) and possess numerous pigment granules (Figure 1u–w), (ii) the availability of growing gametocytes that do not touch erythrocyte envelope (Figure 1s,t), (iii) the pale staining of macrogametocytes (compare Figure 1u with Figure 1v), (iv) the presence of one readily distinguishable vacuole in the majority of the macrogametocytes (Figure 1s), and (v) the subterminal location of nuclei in the majority of macrogametocytes (Figure 1s,u). A detailed description of this pathogen was provided by Valkiūnas et al. [43]. However, the only DNA sequence that was amplified from this bird blood samples was of *Haemoproteus asymmetricus* (lineage hTUPHI01), but the gametocytes of this parasite were not seen in the examined blood films, indicating light parasitemia or sub-patent infection that was hardly detectable under a microscope. 

This bird also was infected by *Leucocytozoon* sp. (lSTUR1) (Figure 1x–ab), which has not been identified to species level yet. The mature gametocytes and host cells (Figure 1z–ab) of this *Leucocytozoon* parasite were similar to *L. dubreuili* (see description above).

Additionally, *Splendidofilaria mavis* was also present in the Song thrush. As in the Eurasian blackbird case, adult worms (three individuals in total) were recovered from the ankle joints. These worms were morphologically indistinguishable from the worms that were seen in the Eurasian blackbird (see description below).

### 3.2. Description of New Lankesterella Species (Eimeriorina, Apicomplexa)

**Figure 2:**
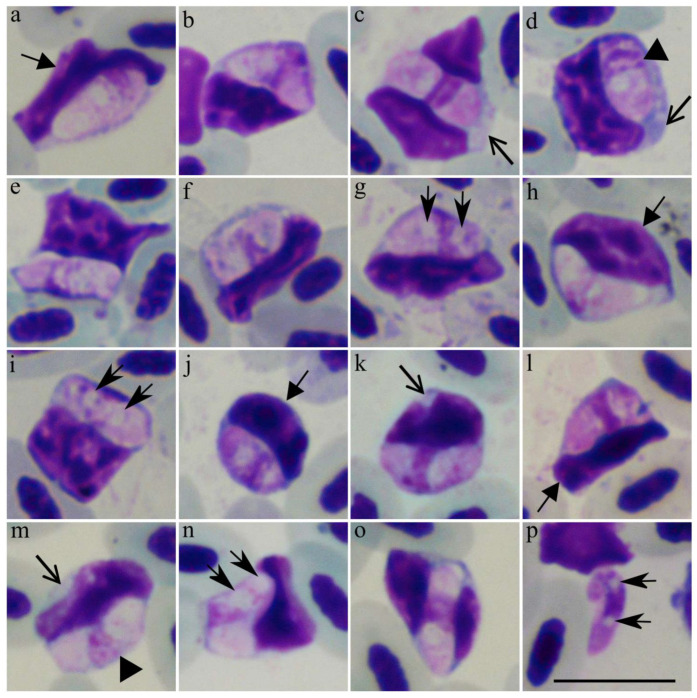
Intracellular (**a**–**o**) and a free (**p**) sporozoites of *Lankesterella bivacuolata* n. sp. (*18S* rRNA lineage LanTurd1) from a Eurasian blackbird (*Turdus merula*). Parasites develop in mononuclear leukocytes, which can be readily distinguished from other leukocytes due to basophilic staining of the cell cytoplasm (**c**,**d**,**l**,**m**). Note that sporozoites markedly deform the host cell nuclei, which were frequently distorted and assumed elongated shapes (**a**,**f**,**g**,**k**,**m**). Two vacuole-like structures were closely appressed to the parasite nuclei (**g**,**i**,**n**,**p**), which are elongate and occupy the entire width of the sporozoites (**d**,**m**). Triangle arrowhead (►), parasite nucleus. Short-barbed arrow (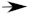), vacuole-like structures. Short triangle arrow (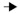), host cell nuclei. Simple wide arrow (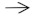), host cell cytoplasm. Scale bar: 10 µm. Methanol fixed and Giemsa-stained blood films.

*Lankesterella bivacuolata* n. sp. (Figure 2, Table 2).

Type host: The Eurasian blackbird (*Turdus merula*) Linnaeus, 1758 (Passeriformes, Turdidae).

Additional hosts: Unknown.

Type locality: Ornithological Station, Ventės Ragas, Lithuania (55°20’28.1” N 21°11’25.3” E).

Site of infection: Mononuclear leukocytes; no other data.

Prevalence: 1 out of 1 examined Eurasian blackbird was infected.

Type specimens: Hapantotype (accession blood film numbers NS49367 and NS49368, adult *Turdus merula,* parasitemia intensity of 4% or 4 parasites per 100 mononuclear leukocytes, Giemsa-stained blood films, Ornithological Station, Ventės Ragas, collected 9 May 2019 by M. Ilgūnas) were deposited in the Nature Research Centre, Vilnius, Lithuania. Parahapantotype (accession numbers G466230 and G466231, other data as for the hapantotype) was deposited in the Queensland Museum, Brisbane, Australia. *Lankesterella* parasites were marked with circles in type preparations. A co-infection with *P. matutinum* (pLINN1), *H. minutus* (hTURDUS2), *Leucocytozoon* spp. (lNEVE01 and lTURMER15), *T. avian*, *T. everetti* group and microfilaria of *S. mavis* are present in the type-material.

DNA sequences: *18S* ribosomal RNA gene lineage LanTurd1 (1030 bp, GenBank accession number OK644714). 

Distribution: This infection has been reported only in the type hosts and type locality as far. There is no sequence with 100% similarity deposited in GenBank. 

Vector: Unknown.

ZooBank registration: the life science identifier (LSID) for the new species *L. bivacuolata* n. sp. is urn:lsid:zoobank.org:pub:15143435-D7D8-4174-ADC7-2A777D8CA9C8.

Etymology: the species name refers to the presence of two small vacuole-like structures in the cytoplasm of sporozoites (Figure 2), which is a characteristic feature of this parasite.

Sporozoites (Figure 2, Table 2) develop in mononuclear leukocytes. The parasites are elongated, with approximately equally rounded ends (Figure 2a,c,h); they were only occasionally seen occupying the entire cytoplasmic space of host cells (Figure 2j,n), and usually, the cytoplasm remnants were well-visible in infected cells (Figure 2c,d,m). The sporozoite cytoplasm stains moderately dark reddish (Figure 2a–p), and it is heterogeneous in appearance. The outline is even. The sporozoites are closely appressed to the infected cell nuclei, which were displaced towards the cell envelope; host-cell nuclei were markedly deformed, frequently assuming an elongated shape (Figure 2a,f,g,l,m), resulting in numerous deformed host-parasite complexes present in blood films. Occasionally, the sporozoites were seen splitting the infected cell nuclei into two portions (Figure 2c,o). Sporozoites length is smaller than the length of host cell nuclei (Figure 2a,d,f,g). The parasite nuclei are elongated, band-like in form; they assume a central or slightly subcentral position and usually occupy the entire width of the sporozoites (Figure 2). Two vacuole-like structures were present in each sporozoite, a characteristic feature of this species. These structures were oval or roundish in shape, relatively small (Figure 2g,i,n; Table 2), and pale-stained; they were frequently seen to be closely appressed to the parasite nuclei and often located on both sides of the nuclei, touching them (Figure 2g,i,p). However, the vacuole-like structures were also seen in other positions in the parasite cytoplasm (Figure 2n). Few free sporozoites were seen in the blood films, and the two vacuole-like structures were also visible (Figure 2p).

Taxonomic remarks: the origin of the host cells (mononuclear leukocytes) can be readily identified in blood films due to the basophilic cytoplasm (Figure 2c,d,m,k), which is not the case in other avian blood cells [44]. The most distinctive morphological feature of *Lankesterella bivacuolata* n. sp. (LanTurd1) is the presence of two small vacuole-like structures, frequently located closely appressed to the parasite nuclei on opposite sides of the nuclei (Figure 2g,i,p). Additionally, the infected host cells are relatively fragile, resulting in their marked deformation in blood films. The host cell nucleus was occasionally seen divided into two portions by the parasite (Figure 2c,o). 

Avian *Lankestrella* parasites were formerly often attributed to the genus *Hepatozoon* [26,45,46,47]. Due to that, tt is logical to compare this new parasite species not only with *Lankesterella* spp., but also with avian *Hepatozoon* species. The presence of a split nucleus was reported in *Hepatozoon pittae*, *Hepatozoon zosteropis*, and *Hepatozoon nephrontis* [45]. Blood stages of *L. bivacuolata* n. sp. can be readily distinguished from these parasites due to several features. First, *H. pittae* [45] is a relatively big parasite, with an average length (10.6 µm), width (3.7 µm) and area (32.1 µm^2^); that is bigger than in *L. bivacuolata* n. sp. (Table 2). Second, the vacuole-like structures are not always present in *H. zosteropis*, and this parasite does not deform infected host cell nuclei [45], but both these features occur in *L. bivacuolata* n. sp. (Figure 2a,c,f). Third, in *H. zosteropis* [45], the average width (4.6 µm) and area (36 µm^2^) are bigger than in *L. bivacuolata*. Fourth, a “cyst-like” wall surrounding the parasite is present in *H. nephrontis*; this parasite also possesses round to ovoid, big-size nuclei, which are located in terminal positions in the sporozoites [45]. None of these characteristics are features of *L. bivacuolata* n. sp. (Figure 2). 

Markedly deformed infected cells were observed in *Lankesterella vacuolata* (LanDeli1) [26]. *Lankesterella bivacuolata* n. sp. (LanTurd1) can be distinguished from this parasite due to the presence of two vacuole-like structures in the cytoplasm of sporozoites (Figure 2g,i,n,p). Furthermore, infected host cells with elongated nuclei (Figure 2a,f,g,k,m) were present in *L. bivacuolata* n. sp. (LanTurd1), but not in *L. vacuolata* (LanDeli1).

**Figure 3:**
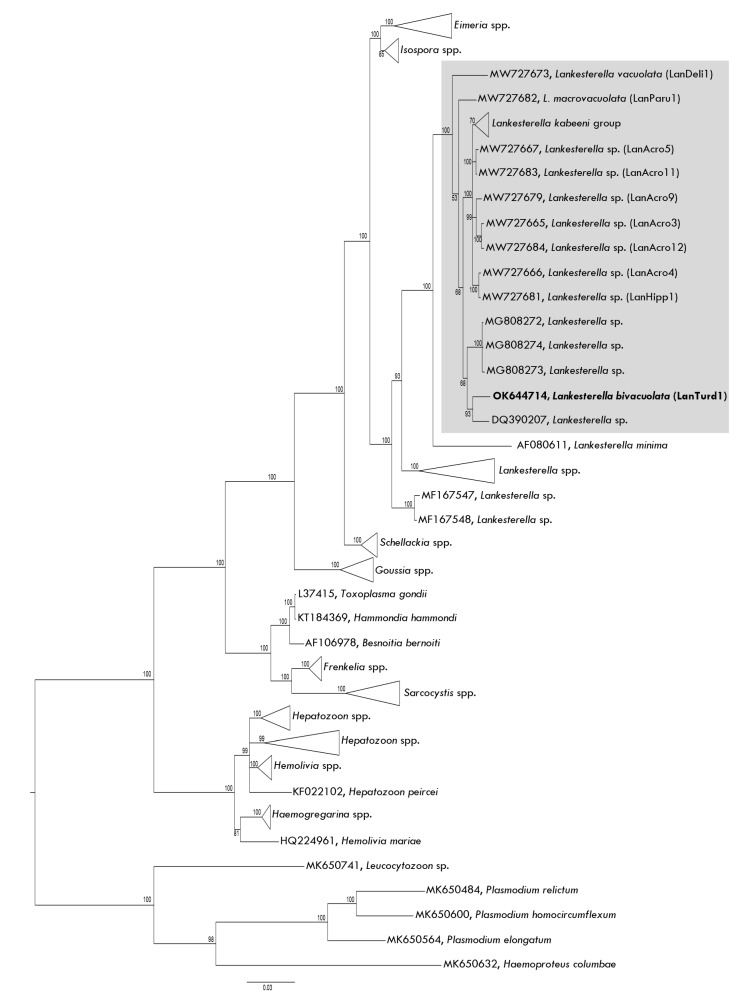
Bayesian phylogenetic inference tree based on the partial *18S* rRNA gene sequences of *Lankesterella* parasites and other closely related Apicomplexa species. The tree was rooted with haemosporidian parasite DNA sequences. *Lankesterella bivacuolata* n. sp. (LanTurd1) found in a Eurasian blackbird (*Turdus merula*) and other avian *Lankesterella* parasites clustered together in one well supported clade (grey box). The name of the new species is given in bold font. GenBank accession numbers are provided for all the sequences. Parasite *18S* rRNA lineage codes of *Lankesterella* parasites are given in parentheses. Nodal support values indicate Bayesian posterior probability.

In phylogenetic analysis, *L. bivacuolata* n. sp. (LanTurd1) is closely related to other avian *Lankesterella* parasites and to *Lankesterella minima,* a parasite of amphibians (Figure 3). The new species is more closely related to *Lankesterella* sp. reported in the Eurasian blue tit *Cyanistes caeruleus* (Figure 3). Phylogenetic analysis supports the conclusion that the avian parasites formerly described as *Hepatozoon* sp., in fact, belong to *Lankesterella*.

### 3.3. Description of Splendidofilaria mavis

**Figure 4:**
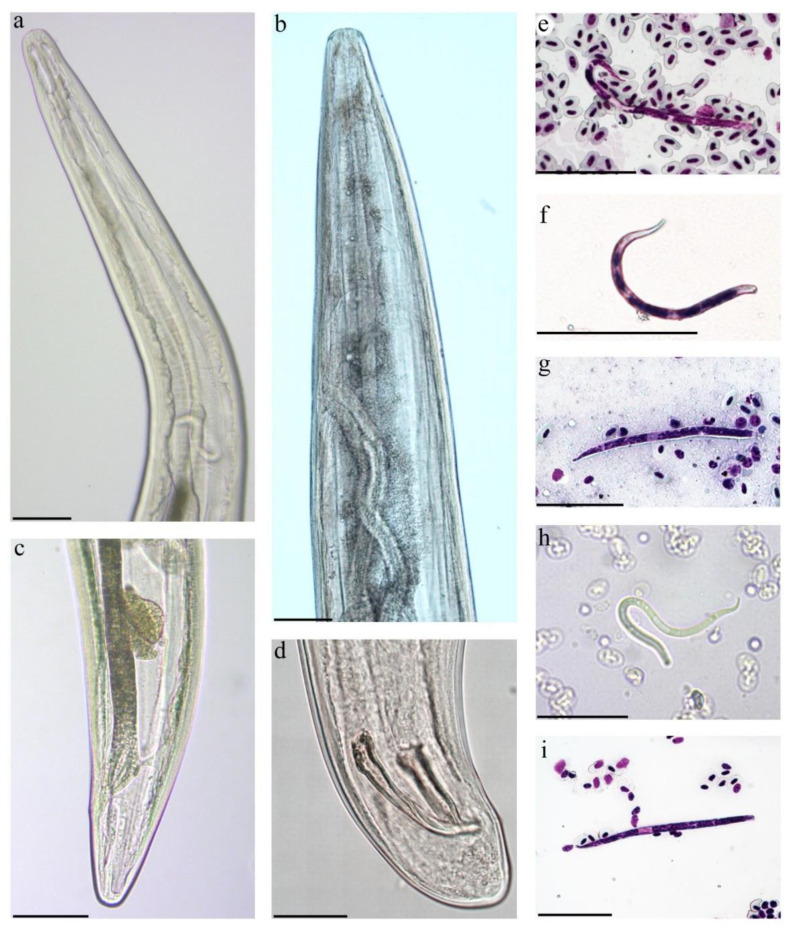
*Splendidofilaria mavis* from a Eurasian blackbird (*Turdus merula*) (**a**–**g**) and a Song thrush (*Turdus philomelos*) (**h**,**i**). Male anterior extremity (alive worm) (**a**). Female anterior extremity (alive worm) (**b**). Female posterior extremity (alive worm) (**c**). Male posterior extremity (ethanol-fixed worm) (**d**). Microfilariae from peripheral blood, Giemsa-stained (**e**,**i**). Microfilariae obtained from female uterus, Giemsa-stained (**f**). Microfilariae from liver blood films, Giemsa-stained (**g**). Alive microfilaria in blood (**h**). Scale bars: 100 µm (**a**,**b**); 50 µm (**c**–**i**).

This study identified microfilariae of *S. mavis* and developed the molecular characterization of this species. Microfilariae that were detected in the blood films of both examined birds had an anterior rounded extremity and tapering rounded posterior extremity with a smooth cuticle (Figure 4e–i). The morphometric characters of the microfilariae from the Eurasian blackbird and the Song thrush are given in Table 3.

No significant difference was discernible in measurements of the microfilaria that were obtained from the lungs or liver. Some specimens in the blood of the lungs and liver had longer cephalic space than the microfilariae in circulatory blood. The microfilariae from the uterus were shorter (75–95 µm) and had a smaller inner body than microfilariae from the blood and other organs but the body shape and fixed-point values (expressed as percentages of the total body) were similar. 

**Figure 5:**
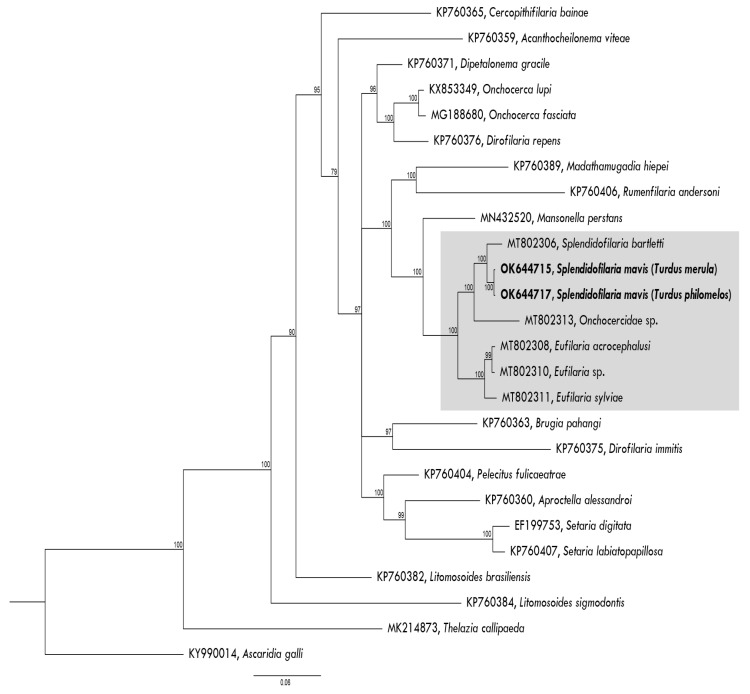
Bayesian phylogenetic inference tree based on partial *28S* nuclear gene sequences of *Splendidofilaria mavis* and other closely related filarioid nematodes. The tree was rooted with *Ascaria galli* DNA sequence. *Splendidofilaria mavis* found in a Eurasian blackbird (*Turdus merula*) and a Song thrush (*Turdus philomelos*) and *Splendidofilaria bartletti* clustered together in a well-supported clade (grey box). Sequences that were obtained in this study are given in bold. The GenBank accession numbers are provided. Nodal support values indicate Bayesian posterior probability.

Adult nematodes are slender with slightly attenuated extremities, males are shorter than females, the cuticle is without bosses, the oral opening is small, the oesophagus is thin, it is not externally divided into muscular and glandular part, the vagina is short, directed posteriorly, the spicules are subequal and dissimilar (Figure 4a–d). The morphometric parameters of adult *S. mavis* in both bird species are similar (Table 4). Although, *S. mavis* as described from the Song thrush had a shorter oesophagus, a vulva that opens further from the anterior extremity and an ovary that turns up father from posterior extremity in comparison to the Eurasian blackbird, but it corresponds with the descriptions of *S. mavis* that was found in Germany [48]. Sequences from adult nematodes of the Eurasian blackbird and the Song thrush clustered in one clade with *Splendidofilaria bartletti* (Figure 5). The sequences of adults and microfilariae coincide.

### 3.4. Circadian Rhythms

#### 3.4.1. Parasites of the Eurasian Blackbird

**Figure 6:**
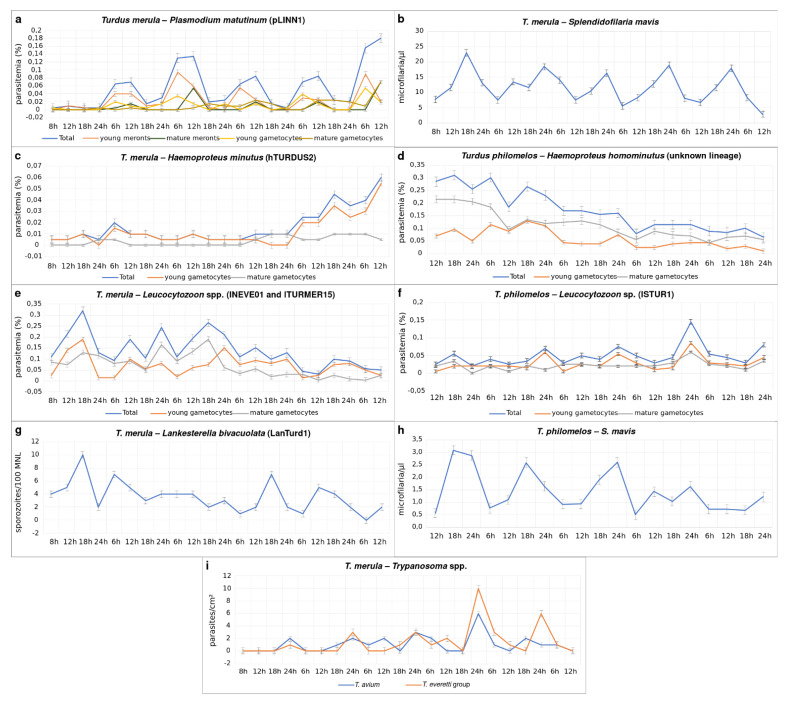
Circadian rhythms of parasites infecting a Eurasian blackbird (*Turdus merula*) (**a**–**c**,**e**,**g**,**i**) and a Song thrush (*Turdus philomelos*) (**d**,**f**,**h**). *Plasmodium matutinum* (cytochrome b lineage pLINN1) (**a**), *Haemoproteus minutus* (hTURDUS2) (**c**), *Haemoproteus homominutus* (unknown lineage) (**d**), *Leucocytozoon* sp. (co-infection of lNEVE01 and lTURMER15) (**e**), *Leucocytozoon* sp. (lSTUR1) (**f**), *Lankesterella bivacuolata* n. sp. (18S rRNA lineage LanTurd1) (**g**), *Trypanosoma avium* and *Trypanosoma everetti* group (**i**), and *Splendidofilaria mavis* (**b**,**h**). Intensity of parasitemia and day hours of the blood sampling were indicated on the ordinate and abscissa, respectively. Vertical bars indicate standard errors.

A 24 h circadian rhythm was observed during *P. matutinum* (pLINN1) infection, with the parasitemia intensity of young meronts and young gametocytes being higher at 6 h, and mature meronts and mature gametocytes were more often seen at 12 h. During the evening (18 h) and night (24 h), the parasitemia intensity of all *P. matutinum* stages was usually lower than in the morning (6 h) and midday (12 h) (Figure 6a). 

In *H. minutus* (hTURDUS2) infection, the intensity of parasitemia was low (<0.01%) during the first four days of the observation and increased to 0.06% only on the fifth day, thus being low during the entire period of observation. No circadian rhythm was observed in this infection (Figure 6c). 

The Eurasian blackbird was co-infected with two *Leucocytozoon* lineages (lNEVE01 and lTURMER15). Even though it is possible to speculate that one of these lineages might be *L. dubreuili* based on the morphology of some of the found gametocytes and their host cells (Figure 1k–o), it is difficult to link the observed gametocytes to a certain lineage because the parasites were similar morphologically. Additionally, many young forms were seen in the blood films (Figure 1e,k). Young *Leucocytozoon* gametocytes are indistinguishable on a species level. This further complicated the linkage of morphological data and lineage information during the examination of circadian rhythms on *Leucocytozoon* species. Thus, the parasitemia intensity was not considered separately for these two lineages. We observed an increase of the parasitemia intensity of young gametocytes every 12 hours, mainly at midday (12 h) and midnight (24 h). For mature gametocytes, the parasitemia peaks were observed always as follow: evening (18 h), midday (12 h), and midnight (24 h) (Figure 6e). 

No circadian rhythm was observed during the studied period for *Lankesterella bivacuolata* n. sp. (LanTurd1) infection, with parasitemia intensity slightly increasing irregularly during a day (Figure 6g).

*Trypanosoma avium* and *T. everetti* group parasites followed the circadian rhythm (Figure 6i), with their parasitemia increasing mainly at midnight (24 h) (Figure 6i). It is interesting to mention that parasitemia intensity also increased a few times in other times of the day during the observation period, i.e., *Trypanosoma avium* had one peak at midday (12 h) and one in the evening (18 h), while parasitemia of *T. everetti* group parasite had one peak at midday (12 h).

*Splendidofilaria mavis* microfilariae were present in the blood permanently (Figure 6b), however, it was possible to observe its circadian rhythm. Mainly, the lowest parasitemia intensity was in the morning (6 h) and at midday (12 h), while the biggest parasitemia was recorded in the evening (18 h) and night (24 h) (Figure 6b).

#### 3.4.2. Parasites of the Song Thrush

In *H. homominutus* (unknown lineage) infection, the intensity of parasitemia was around 0.3% at the beginning of the observation, and it decreased along the observation days to 0.1%. As for *H. minutus,* no circadian rhythm was observed in this infection (Figure 6d).

The song thrush had a single *Leucocytozoon* (lSTUR1) infection, and in this case, the peaks of parasitemia of young gametocytes were reported at midnight (24 h). Interestingly, the intensity of mature gametocytes was constant throughout the experiment, with small peaks in the morning (6 h) and the evening (18 h) (Figure 6f).

The circadian rhythms of *S. mavis* in the Song thrush were the same as in the Eurasian blackbird (Figure 6h).

## 4. Discussion

The key results of this study are (i) the description and molecular characterization of *Lankesterella bivacuolata* n. sp. (lineage LanTurd1) infecting a Eurasian blackbird; (ii) molecular characterization of the filarioid nematode *S. mavis* infecting Turdidae birds; (iii) the first description of the circadian rhythm of *Plasmodium matutinum* (pLINN1) at the lineage level; (iv) the first provided information about the circadian rhythm of *Haemoproteus, Lankesterella* and *S. mavis* parasites; and (v) evidence that circadian rhythms of *P. matutinum* (pLINN1) and *S. mavis* maintain during co-infection with several different blood parasites. These findings are discussed below.

### 4.1. Description of New Lankesterella Parasite 

A recent study by Chagas et al. [26] reviewed available information about avian *Lankesterella* blood parasites and pointed out that some avian blood parasites, which have been formerly attributed to the genus *Hepatozoon,* in fact, belong to *Lankesterella* genus [46,47,49]. Our phylogenetic analysis supports this conclusion due to the close clustering of all currently available avian *Lankesterella* lineages, including the lineage LanTurd1 of the new species (Figure 3). We also extend information about the diversity and phylogenetic relationships of these neglected avian parasites by describing *L. bivacuolata* n. sp. (LanTurd1), which is readily distinguishable from all described *Lankesterella* species due to the presence of two vacuole-like structures in the sporozoites (Figure 2g,i,n,p). 

The available information that is based on the detection of *18S* rRNA lineages in avian hosts indicates that *Lankesterella* parasites likely are specific to birds on their genera levels [26]. *Lankesterella bivacuolata* n. sp. (LanTurd1) was also found in a Eurasian blackbird in the Labanoras Forest (55°12′25.77″ N, 25°55′26.47″ E), Lithuania (C. Chagas personal communication, data not shown). It is important to mention that the complete life cycle and vectors of avian *Lankesterella* parasites remain unknown. More studies, particularly those targeting potential vectors, transmission, life cycles, and analyses of other genes should be encouraged to better understand *Lankesterella* spp. biology and their role in the wild. This study provides information for morphological identification and molecular barcoding of *L. bivacuolata* n. sp. (LanTurd1)*,* the parasite of the Eurasian blackbird.

### 4.2. Description and Molecular Characterization of Splendidofilaria mavis

In spite of much former research on morphology and taxonomy of filarioid nematodes [31,50,51,52,53,54], the molecular characterization of only a few avian filarioid nematodes is available [4]. This is unfortunate because molecular barcoding provides opportunities to detect the parasites at the microfilariae stage in circulation avoiding bird dissection and thus might remarkably extend sampling and data collection in wildlife. We contribute to the development of barcoding of Onchocercidae nematodes by providing DNA sequences of *S. mavis*. This study extends information about the morphology on adult worms of *S. mavis* and the microfilariae of this parasite, which were found in two different species of thrushes (Turdidae). This provides an opportunity to develop the phylogenetic analysis on relationships of avian wildlife filarioid nematodes (Figure 5). Future research on these parasites using morphological and molecular approaches in parallel should be encouraged. Such approaches open new opportunities for more reliable species identification and would extend screening of birds for Onchocercidae nematodes using analysis of blood samples. 

The onchocercidian parasite *S. mavis* was first found in the redwing (*Turdus iliacus,* synonym *Turdus musicus*) in the United Kingdom [30]. Then, this nematode was detected in different species of thrushes in Germany, Austria, and Poland [48,55]. The morphometric parameters of *S. mavis* that were described in these countries and our measurements are almost identical. Differently from earlier descriptions, our study shows that the vagina can be longer—up to 128 µm instead of 50 µm reported before, and the left spicule can also be longer—up to 116 µm instead of 90 µm. The microfilariae named *S. mavis* were found in thrushes in Spain, France, and Czech Republic [53,56,57], and also reported in other passerine birds, such as the Hawfinch (*Coccothraustes coccothraustes*) and the European robin (*Erithacus rubecula*) [53,58]. These findings indicate that *S. mavis* might not be strictly specific to the final host. However, there was no convincing evidence that these microfilariae certainly belong to *S. mavis*. The development of molecular markers for species identification are crucial for future studies on filarioid nematodes that are found in different host species and present at different stages of development because molecular barcoding will minimize misidentifications.

It is worth noting that *Splendidofilaria* (synonym *Ornithofilaria*) *bohmi* was found in the Mistle thrush (*Turdus viscivorus*) in Europe [59]. According to review by Sonin [54], the morphological differences between this parasite and *S. mavis* are insignificant; these parasites slightly differ from each other due to the size of spicules. Additional material is needed for a conclusion about validity of the name *S. bohmi.* Our morphological analysis shows that the length of spicules in *S. mavis* overlaps with those in *S. bohmi* (left spicules 103 µm, 116 µm, and 102–125 µm, respectively; right spicules 82 µm, 91 µm, and 86–110 µm, respectively). These two species differ only due to (i) the different distance from anterior extremity to vulva (466–795 µm in *S. mavis* and 360–380 µm in *S. bohmi*) and (ii) the shape of microfilariae. Supperer [59] published a good-quality description and pictures of *S. bohmi* microfilariae, which were over 155 µm in length, and their posterior part was slightly narrowed and broadly rounded. Our study of microfilariae and the microfilariae description given by Gönnert [48] showed that the microfilariae are shorter (73–143 µm). Furthermore, according to our investigation, the posterior extremity of *S. mavis* microfilariae are tapering rounded, almost pointed, that distinguish them from *S. bohmi*, indicating possible validity of both names. The shape of the microfilariae that were found in France and Czech Republic [53,56,58] was more similar to *S. bohmi* microfilariae because their posterior has a broadly rounded end than in *S. mavis*. Despite the differences in the shape of microfilariae, the morphology of the adult worms was almost identical. To prove that these parasites are different species, further taxonomy studies are needed, ideally by a combination of morphological features and molecular markers.

### 4.3. Circadian Rhythms of Blood Parasites during Co-Infections

Considering that over 260 avian haemosporidian parasites are described and likely many more exist [60], the available information about their circadian rhythms is certainly insufficient. Most of the studied avian parasite species belong to *Plasmodium*, but the available information deals mainly with the circadian rhythms of erythrocytic merogony, with rare exceptions when diurnal fluctuation in gametocytes intensity was considered [3,16]. Circadian rhythms have never been investigated in *Haemoproteus* infections, and information is available only about circadian rhythms of two *Leucocytozoon* parasites, i.e., *Leucocytozoon simondi* and *L. smithi* [3]. Avian *Lankesterella* are the most poorly investigated parasites, and this study provided the first information on their circadian rhythms. Daily parasitemia cycles in *Trypanosoma* parasites are available mainly for species that infect humans, those with zoonotic potential and that are economically important [21]. A few studies address this issue in *Trypanosoma* parasites of amphibians [61]. Filarioid nematodes are relatively well-investigated with regard to circadian rhythms, with many studies conducted, even though they are also more focused on species that infect humans and other mammals [21,62,63]. 

It is important to note that this study does not propose any conclusions on circadian rhythms in each parasite group because it is limited by a few observations in only two avian hosts. Additionally, co-infection was present in both studied hosts, raising a question about the possible influence of parasites on each other. However, we used this opportunity to describe the available data to get maximum information from these birds which were dissected for research. Data about the circadian rhythms of avian blood parasites during co-infections are absent, thus might provide ideas for further experimental investigations. Below, we discuss the main information that was obtained about circadian rhythms by different parasite groups.

#### 4.3.1. *Plasmodium* Infections

The periodicity of several avian *Plasmodium* species has been investigated. Most of them have an erythrocytic merogony cycle of 24 h, with the peak of meront maturation varying in different species along the day. For instance, *P. matutinum*, *P. giovannolaia*, and *P. elongatum* have the biggest intensity of mature meronts in the morning; in *P. cathemerium* and *P. circumflexum* infections, a peak was seen in the evening; and in *P. gabaldoni, P. nucleophilum*, and *P. homocircumflexum* (lineage pCOLL4), the intensity of meronts was higher at midday [3,16]. In *P. gallinaceum* and *P. relictum*, an erythrocytic merogony cycle of 36 h was reported [3]. In this study, we determined the circadian rhythms of the lineage pLINN1 of *P. matutinum* for the first time; it was close to 24 h, as formerly reported for an unknown lineage of the same parasite species [64], with a bigger intensity of mature meronts seen in the morning (6 h) and a bigger intensity of mature gametocytes at midday (12 h) (Figure 6a). 

Previous studies have reported that *Plasmodium* sp. parasitemia intensity and the parasitemia dynamics might differ in different avian hosts [65,66] and also during co-infections of two *Plasmodium* species [67]. In the present study, the circadian rhythm of the mature meronts remained the same in *P. matutinum* (pLINN1), as indicated for an unknown lineage of *P. matutinum* by Garnham [64] and Valkiūnas [3]. Importantly, our study showed that the circadian rhythms of mature meronts remained even during co-infections of several species and lineages of haemosporidian and other blood parasites, indicating the relatively stable pattern of the periodicity in *P. matutinum*. This avian malaria parasite is widespread in thrushes [68] and can be recommended for experimental studies on circadian rhythms of avian malaria infections with rapid (24 h) cycles of erythrocytic merogony. 

#### 4.3.2. *Haemoproteus* Infections

Only gametocytes are present in the circulation during *Haemoproteus* infection. No circadian rhythm was discernible during parasitemia of both studied *Haemoproteus* species, i.e., *H. minutus* (hTURDUS2) (Figure 1f–j) and *H. homominutus* (the parasite was identified only through microscopic examination) (Figure 1s–w). It is interesting to note that *H. minutus* (hTURDUS2) parasitemia increased throughout the course of this study (Figure 6c), but reverse dynamic of parasitemia was seen during *H. homominutus* infection. Mainly, the parasitemia intensity decreased during the observation (Figure 6d). It is possible to speculate that stress conditions might influence the increase of *H. minutus* (hTURDUS2) parasitemia in the Eurasian blackbird. The stress in this host might be particularly severe due to the presence of seven other co-infecting blood parasites. It is known that capturing wild animals leads to an increase in corticosteroid levels, a hormone indicative of physiological stress. This increase can result in an impairment of the immune response of the infected animals and might lead to a higher parasitemia [69,70]. At the same time, the studied birds had access to food *ad libitum*, which might have increased the availability of resources that they could use to mount an immunological response and reduce parasitemia [71]. That might be the case in the Song thrush. This bird was infected with *H. homominutus* and co-infected with only two other parasite species, so might be under less parasite-induced stress pressure, resulting in a decrease of parasitemia during this study (Figure 6d). The role of immunological responses on the parasitemia of haemosporidian parasites remains insufficiently studied, particularly in wildlife. 

This study further points out difficulties in haemosporidian parasite diagnostics during co-infections. Mainly, even though the intensity of parasitemia of gametocytes of *H. homominutus* were intense, and the parasite was readily recognizable in blood films of the Song thrush (Figure 1v,w), we did not recover this parasite sequence in the samples. However, the sequence *H. asymmetricus* (hTUPHI01) was detected. These two genetically closely related parasites often parasitize thrushes; they are sometimes reported in co-infection due to readily distinguishable gametocytes [72]. It is possible to speculate that the studied Song thrush was co-infected with *H. homominutus* and *H. asymmetricus*, but the latter infection was latent. A template for *H. asymmetricus* (hTUPHI01) DNA amplification might come from the remnants of the tissue stages, which are difficult to visualize on blood films, but not from gametocytes. The presence of haemosporidian co-infections has been frequently reported in wild birds [6,7,8,9], and such infections even predominate in some bird populations in Europe [10]. Conventional PCR-based methods are not always able to diagnose these co-infections [11,73] because general primers might have a better match to certain DNA sequences. This study further highlights the importance of both microscopy examination and PCR-based tools in wildlife haemosporidian research [68]. 

#### 4.3.3. *Leucocytozoon* Infections

Information about circadian rhythm is available only for two *Leucocytozoon* species, i.e., *Leucocytozoon simondi* and *Leucocytozoon smithi*, the parasites of domestic ducks and turkeys, respectively. Only gametocytes are present in the circulation in *Leucocytozoon* infections and parasitemia usually increase during the daytime [3]. Information about circadian rhythms of *Leucocytozoon* species infecting thrushes and other wild birds is absent. 

In this study, the Eurasian blackbird was co-infected with two genetic lineages of *Leucocytozoon* sp., in which gametocytes were hardly distinguishable morphologically and were unidentified to species levels. The identification of *Leucocytozoon* parasites using morphological characteristics of their blood stages (gametocytes) is challenging due to the limited number of valuable taxonomic morphological characters, which are often similar in many described species. Additionally, gametocytes and host cells of these parasites are fragile and often are deformed in blood films (for example, see Figure 1z,ab). As a result, we were unable to identify *Leucocytozoon* species during co-infections which were determined by the PCR-based method. In other words, the circadian rhythm was not determined for each *Leucocytozoon* parasite lineage separately. However, we observed that young gametocytes were present every 12 hours in the blood, while mature gametocytes can be found at different times of the day (Figure 6e). 

The *Leucocytozoon* sp. (lSTUR1) was present in a single infection in the Song thrush. During this infection, young gametocytes were more frequently observed in the evening (18 h) and night (24 h), and mature gametocytes were predominant every 12 hours, in the evening (18 h) and morning (6 h) (Figure 6f). 

The available data on parasitemia dynamics of *Leucocytozoon* species suggest that different parasite species and/or lineages might have different circadian rhythms. This might prevent infection of the same individual vector with several different lineages of related parasites of the same genus, and thus might contribute to minimizing competition during the sporogony process. It is interesting to mention that *Leucocytozoon* parasites are transmitted by Simuliidae blackflies [3]. These blood-sucking insects are particularly active during the daytime [74]. The presence of mature gametocytes in the circulation during this time would be beneficial for transmission and should be evolutionary preferable.

#### 4.3.4. *Lankesterella* Infection

Avian *Lankesterella* parasites remain a neglected group of pathogens with numerous unclear issues in their basic biology, particularly related to their pathogenicity and transmission [26,46,47,49]. This is the first study that has addressed the circadian rhythm of *Lankesterella* parasites which are probably transmitted by different mosquito species [26]. 

We did not observe any circadian rhythm in the parasitemia intensity of *L. bivacuolata* n. sp. (LanTurd1) during the studied period (Figure 6g). Further studies are needed for a better understanding of whether the circadian rhythm occurs in other species of *Lankesterella.*

Only the final stage of the life cycle (sporozoites) is present in bird blood, and the vectors serve mainly as a reservoir of infection, which does not develop in insects. Birds are likely to get infected mainly by eating infected insects in which the parasite can persist over a month after the initial infection [26]. Circadian rhythms might be of biologically low value for the transmission of such parasites which might be not specific to certain vector species resulting in possible transmission during different times of the day. However, more studies are needed for a better understanding of the vector biology of *Lankesterella* spp. 

#### 4.3.5. *Trypanosoma* Infections

Circadian rhythms were recognizable in parasitemia of both *T. avium* and *T. everetti* group parasites with increases of parasitemia intensity at night close to 24 h (Figure 6i). These parasites might be transmitted by *Culicoides* biting midges [27,75], which are more active during the night as well. *Trypanosoma avium* was also reported to be transmitted by Simuliidae insects [76]. These insects are more active during the day, however, only a few peaks of parasitemia were seen during *Trypanosoma* infections during the daytime in the present study. This might suggest that Simuliidae species may not be the main vectors of the examined parasites. *Culicoides* biting midges are more active during the sunset and the sunrise in Europe [77]. An increase of parasitemia close to the night period should be beneficial for transmission. It is interesting to note that an increase of parasitemia during the night was reported in some other *Trypanosoma* species. For example, *Trypanosoma lewisi, T. duttoni*, and *T. congolense*, which parasitize rodents [78,79]. However, this is certainly not a general pattern in *Trypanosoma* species. For example, parasitemia of *T. rotatorium*, a parasite of frogs, increases during the daytime close to 12 h [61]. 

Parasitemia of avian trypanosomes is usually low in birds [80]. The obtained information about the daytime peaks of parasitemia of common *T. avium* and *T. everetti* group parasites is helpful for the selection of donor hosts for the pathogen strains isolation and experimental research.

#### 4.3.6. Microfilariae Infections

This study shows that *S. mavis* microfilariae exhibited a nocturnal periodicity in both studied birds. Judging from the daily parasitemia intensity of microfilariae, the best time for the transmission of this pathogen should be close to the night when numerous parasites were seen in the circulation, suggesting a greater probability to infect vectors. Nevertheless, microfilariae were observed in the circulation during the entire day (Figure 6b,h). Hence, vectors can also have access to infection during the entire day. The available knowledge shows that the circadian rhythms of microfilariae depend markedly on the parasite and host species. The parasitemia peaks can be nocturnal, diurnal, or even irregular. An increase of microfilariae parasitemia usually coincides with a period of maximum activity of the local arthropods vector species [81]. This is particularly well-studied in the human parasite *Loa loa*, where microfilariae exhibit a marked diurnal parasitemia periodicity and this is well-associated with the time of maximum activity of its intermediate hosts, the deer flies *Chrysops silacea* and *Chrysops dimidiate* [82]. Meanwhile, the microfilariae of *Wuchereria bancrofti*, another pathogenic human parasite, exhibit a marked nocturnal periodicity and are transmitted by mosquitoes of different genera, which are active at night (*Aedes, Anopheles, Culex*, and *Monsonia*). 

Few studies are addressing the circadian parasitemia rhythms of avian filarioid nematodes. These are: *Cardiofilaria nilesi*, *Chandlerella striatospicula*, *Splendidofilaria picacardina*, and *S. fallisensis* [21,63]. The circadian rhythm of *S. picacardina* microfilariae in the Black-billed magpie (*Pica hudsonia*) exhibited nocturnal periodicity, as also was the case with *S. mavis* in our study. However, the biggest parasitemia intensity of microfilariae was seen during the daytime in *S. fallisensis*, a parasite of North American ducks [21]. Females of black flies feed on blood during the day [74], therefore it is not surprising that the peak of *S. fallisensis* microfilaria in the blood also occurred during this period of the day. The vectors of *S. picacardina* and *S. mavis* remain unknown. Many Onchocercidae species of mammals exhibit a nocturnal peak of microfilariae parasitemia and their vectors are species of *Aedes*, *Anopheles*, *Armigeres*, *Coquillettidia*, *Culex*, *Culicoides*, *Mansonia*, and *Ochlerotatus* mosquitoes [28,83,84,85,86,87,88]. This indicated that ornithophilic mosquitoes from these genera likely can be the vectors of nocturnal *Splendidofilaria* species as well.

## 5. Conclusions

This study described one new species of an avian blood parasite, *Lankesterella bivacuolata* n. sp. (lineage LanTurd1) and provided the molecular characterization and detailed morphological description of the filarioid nematode *Splendidofilaria mavis*, a common parasite of passerine birds. Importantly, microfilariae of this nematode were not only described and illustrated, but also characterized molecularly. Both the molecular characterization and the microfilariae description open new opportunities for prominent parasite sampling that is aimed at a better understanding of the biology of filarioid nematodes using harmless-for-host methods. The molecular characterization of both these pathogens expand the opportunities for phylogenetic research on blood parasites of related genera.

The information provided about circadian rhythms suggests directions for the planning of future targeting experimental observations on this insufficiently understood subject. It was shown that clear circadian rhythms of parasitemia are maintained in *Plasmodium matutinum* (pLINN1) and *S. mavis* infections even during the co-infection of numerous different parasites, thus being relatively insensitive for the influence of other pathogens that are present in same host and indicating possible essential importance of the parasitemia fluctuation in these parasites’ biology and transmission. 

This was the first study that addressed the daily variation in parasitemia of avian blood parasites belonging to *Haemoproteus, Lankesterella* and *S. mavis*. Circadian rhythms do not occur in *Haemoproteus* and *Lankesterella* parasite species, at least during co-infections. 

## Figures and Tables

**Table 1:** Methodology used to determine parasitemia intensity and PCR-protocol applied for DNA amplification of blood parasites found in the Eurasian blackbird (*Turdus merula*) and the Song thrush (*Turdus philomelos*).

Parasite Genus	Parasitemia Intensity Determination	PCR-Protocol	Gene Amplified
*Plasmodium*	Infected cells on 20,000 erythrocytes [24]	[25]	Cytochrome *b* (*cytb*)
*Haemoproteus*	Infected cells on 20,000 erythrocytes [24]	[25]	*cytb*
*Leucocytozoon*	Infected cells on 20,000 erythrocytes [24]	[25]	*cytb*
*Lankesterella*	Infected cells on 100 mononuclear leukocytes [26]	[26]	*18S* ribosomal RNA (*18S*)
*Trypanosoma*	Trypomastigotes seen in 1 cm^2^ of blood film	[27]	*18S*
*Splendidofilaria*	Microfilariae seen 1 µL of blood	[4]	*28S* nuclear (*28S*) and cytochrome oxidase I (*coxI*)

**Table 2:** Morphometry of sporozoites of *Lankesterella bivacuolata* n. sp. (*18S* rRNA lineage LanTurd1). All the measurements are given in micrometres.

Feature	Measurements (n = 21) ^a^
Sporozoite	
Length	8.2–9.9 (9.1 ± 0.4) ^b^
Width	2.6–4.0 (3.4 ± 0.4)
Area	21.9–29.3 (25.7 ± 2.2)
Sporozoite nucleus	
Length	1.0–2.5 (2.0 ± 0.4)
Width	1.5–3.6 (2.7 ± 0.6)
Area	2.7–5.4 (3.9 ± 0.7)
Sporozoite vacuole-like structure	
Length	1.0–2.0 (1.5 ± 0.2)
Width	1.4–2.9 (2.2 ± 0.4)
Area	1.2–3.8 (2.8 ± 0.7)

^a^ Number of measured parasites. ^b^ Minimum and maximum values are provided, followed in parentheses by the arithmetic mean and standard deviation.

**Table 3:** Measurements of *Splendidofilaria mavis* microfilariae from the blood, lungs, and uterus form the Eurasian blackbird (*Turdus merula*) and the Song thrush (*Turdus philomelos*).

	*Turdus merula*						*Turdus philomelos*			
Parameter	Blood (n = 25)		Lungs (n = 20)		Uterus (n = 7)		Blood (n = 10)		Lungs (n = 17)	
	MC	%	MC	%	MC	%	MC	%	MC	%
Length	83–120	-	86–143	-	75–95	-	96–124	-	73–113	-
Maximum width	4–6	-	5–6	-	3–5	-	4–6	-	4–6	-
Inner body length	6–14	-	5–15	-	2–5	-	6–12	-	4–13	-
Cephalic space	1–4	-	1–7	-	2–6	-	1–3	-	1–7	-
Distance from AE to:										
nerve ring	16–26	18–24	17–34	19–28	18–25	22–26	18–27	17–24	16–27	18–25
excretory pore	24–47	28–35	26–49	30–35	24–31	30–36	31–40	29–35	22–39	26–36
inner body anterior end	44–70	47–56	44–81	46–57	41–53	52–56	53–69	51–56	39–62	48–55
inner body posterior end	50–85	57–65	51–90	55–65	44–58	55–61	60–79	58–64	43–73	58–67
anal pore	67–108	81–87	70–119	80–85	60–78	79–82	82–105	83–87	59–96	80–88

MC, morphometric characteristics are given in micrometres; %, values of fixed points expressed in percentage; (-), not evaluated; AE, anterior extremity.

**Table 4:** Measurements of adult *Splendidofilaria mavis* from the Eurasian blackbird (*Turdus merula*) and the Song thrush (*Turdus philomelos*) that were examined in this study. All measurements are given in micrometres, except for body length, which is given in millimetres.

Feature	*Turdus merula*		*Turdus philomelos*	
	Male (n = 1)	Female (n = 4)	Male (n = 1)	Female (n = 1)
Body length	8	11–17	8	15
Maximum width	345	320–403	253	380
Oesophagus length	890	1010–1026	729	791
Vagina length	-	108–128	-	82
Left spicule length	116	-	103	-
Right spicule length	82	-	91	-
Nerve ring from AE	118	128–182	118	126
Vulva from AE	-	466–646	-	795
Testes from AE	547	-	546	-
Ovary from PE	-	86–239	-	447
Cloaca/Anus from PE	53	73–110	70	153

AE, anterior extremity; PE, posterior extremity; (-) not evaluated.

## Data Availability

The data that are presented in this study are in GenBank database (https://www.ncbi.nlm.nih.gov/genbank/, accessed on 27 October 2021) (accession numbers: *P. matutinum* pLINN1, OK646331; *H. minutus* hTURDUS2, OK646332; *H. asymmetricus* hTUPHI01, OK721103, *Leucocytozoon* sp. lNEVE01, OK646333; *Leucocytozoon* sp. lTURMER15, OK646334; *Leucocytozoon* sp. lSTUR1, OK646335; *L. bivacuolata* n. sp., OK644714; *T. avium*, OK605056; *S. mavis 28S*, OK644715-OK644718; and *S. mavis coxI*, OK631737-OK391740).
